# Aggravation of Chronic Stress Effects on Hippocampal Neurogenesis and Spatial Memory in LPA_1_ Receptor Knockout Mice

**DOI:** 10.1371/journal.pone.0025522

**Published:** 2011-09-29

**Authors:** Estela Castilla-Ortega, Carolina Hoyo-Becerra, Carmen Pedraza, Jerold Chun, Fernando Rodríguez De Fonseca, Guillermo Estivill-Torrús, Luis J. Santín

**Affiliations:** 1 Departamento de Psicobiología y Metodología de las CC, Universidad de Málaga, Campus de Teatinos, Málaga, Spain; 2 Unidad de Investigación, Fundación IMABIS, Hospital Regional Universitario Carlos Haya, Málaga, Spain; 3 Department of Molecular Biology, Dorris Neuroscience Center, The Scripps Research Institute, La Jolla, California, United States of America; University of Queensland, Australia

## Abstract

**Background:**

The lysophosphatidic acid LPA_1_ receptor regulates plasticity and neurogenesis in the adult hippocampus. Here, we studied whether absence of the LPA_1_ receptor modulated the detrimental effects of chronic stress on hippocampal neurogenesis and spatial memory.

**Methodology/Principal Findings:**

Male LPA_1_-null (NULL) and wild-type (WT) mice were assigned to control or chronic stress conditions (21 days of restraint, 3 h/day). Immunohistochemistry for bromodeoxyuridine and endogenous markers was performed to examine hippocampal cell proliferation, survival, number and maturation of young neurons, hippocampal structure and apoptosis in the hippocampus. Corticosterone levels were measured in another a separate cohort of mice. Finally, the hole-board test assessed spatial reference and working memory. Under control conditions, NULL mice showed reduced cell proliferation, a defective population of young neurons, reduced hippocampal volume and moderate spatial memory deficits. However, the primary result is that chronic stress impaired hippocampal neurogenesis in NULLs more severely than in WT mice in terms of cell proliferation; apoptosis; the number and maturation of young neurons; and both the volume and neuronal density in the granular zone. Only stressed NULLs presented hypocortisolemia. Moreover, a dramatic deficit in spatial reference memory consolidation was observed in chronically stressed NULL mice, which was in contrast to the minor effect observed in stressed WT mice.

**Conclusions/Significance:**

These results reveal that the absence of the LPA_1_ receptor aggravates the chronic stress-induced impairment to hippocampal neurogenesis and its dependent functions. Thus, modulation of the LPA_1_ receptor pathway may be of interest with respect to the treatment of stress-induced hippocampal pathology.

## Introduction

Adult hippocampal neurogenesis is a form of structural plasticity that occurs in the dentate gyrus (DG) of the hippocampus. Newly born precursor cells originate from stem cells in the subgranular zone (SGZ) of the DG and migrate to the granular cell layer. Here, they integrate into the neuronal circuitry of the DG as granule neurons [1]–[3]. Though controversial, several studies have implicated newly generated neurons in both hippocampal function and forms of hippocampal-dependent memory, such as spatial memory, spatial pattern separation and contextual fear memory [4]–[6]. Many factors can influence hippocampal neurogenesis in adulthood [7], . In this regard, the deleterious consequences of chronic exposure to stress for both hippocampal neurogenesis and hippocampal-dependent behaviour is well known [9]–[11]. In general, chronic stress reduces the proliferation, survival and the capacity for neuronal differentiation of newly born cells [10], [12]–[15]. Chronic stress has also been shown to dysregulate apoptosis in the DG [16], [17]. It is believed that a decline in hippocampal neurogenesis markedly contributes to the behavioural consequences of chronic stress, causing cognitive and emotional psychopathology [18]–[20].

It has recently been reported that lysophosphatidic acid (LPA, 1-acyl-2-*sn*-glycerol-3-phosphate) is involved in adult neurogenesis in the mammalian brain. LPA is a phospholipid synthesised from the cell membrane and acts as an intercellular signalling molecule through 6 different G-protein-coupled receptors (LPA_1-6_) [21]–[29]. The LPA_1_ receptor not only mediates the proliferation, migration and survival of neural progenitor cells during brain development [25], [28], [30] but also plays an important role in adult hippocampal neurogenesis. In this regard, LPA_1_ is expressed in hippocampal progenitor cells, where it is involved in neural differentiation [31], [32] and in adult hippocampal neurons where it promotes synaptic modifications [33]. Mice lacking the LPA_1_ receptor demonstrate defective proliferation and maturation of newly born neurons and a blunted increase in cell proliferation and survival in response to environmental enrichment [34]. Furthermore, these hippocampal deficits were accompanied by behavioural impairments, such as impaired spatial memory retention, altered exploration and increased anxiety-like responses [35], . Certain behavioural alterations in the cognitive and emotional domains, such as spatial memory impairments and enhanced anxiety-like behaviours, are severely affected when hippocampal neurogenesis is ablated [4], [5], [8], [37].

On the basis of the reported role of the LPA_1_ receptor in adult neurogenesis and the effect of chronic stress on this form of structural plasticity, we hypothesised that the LPA_1_ receptor may also regulate the impact of chronic stress on both SGZ neurogenesis and hippocampus-related behaviours. If this is true, the absence of the LPA_1_ receptor would confer enhanced vulnerability to chronic stress. To address this issue, we assessed hippocampal structure, cell proliferation, apoptosis, new neuron's maturation and cell survival in LPA_1_-null mice and their wild-type (WT) littermates under both basal conditions and following chronic restraint. Basal corticosterone levels were also measured in control and stressed mice of both genotypes. In addition, hippocampal-related behaviour was examined using the hole-board test, a behavioural task that allows for the assessment of exploratory behaviour, anxiety and both spatial reference and working memory [35].

## Methods

### Animals

The generation and characterisation of maLPA_1_-null mice has been described previously [30]. An LPA_1_-null mouse colony, termed maLPA_1_ after the *laga* variant of the LPA_1_ knockout, was spontaneously derived during the original colony [38] expansion by crossing heterozygous foundation parents (maintained in the original hybrid C57BL/6J ×129X1/SvJ background). Intercrosses were performed with these mice and were subsequently backcrossed for 20 generations with mice generated within this mixed background. MaLPA_1_-null mice carrying the *lpa*_1_ deletion were born at the expected Mendelian ratio and survived to adulthood. Targeted disruption of the *lpa*_1_ gene was confirmed by genotyping [38], and immunochemistry confirmed the absence of LPA_1_ protein expression.

All experiments were conducted on approximately three-month-old age-matched male littermates from the WT and maLPA_1_-null homozygous (NULL) genotypes. Mice were singly housed on a 12-h light/dark cycle (lights on at 07:00 a.m.). Water and food were provided ad libitum. All procedures were performed in accordance with European animal research laws (European Communities Council Directives 86/609/EEC, 98/81/CEE and 2003/65/CE; Commission Recommendation 2007/526/EC) and the Spanish National Guidelines for Animal Experimentation and the Use of Genetically Modified Organisms (Real Decreto 1205/2005 and 178/2004; Ley 32/2007 and 9/2003). All animal procedures were approved by the Institutional Animal Care and Use Committee of Malaga University (approval ID SEJ2007- 61187).

**Figure 1:**
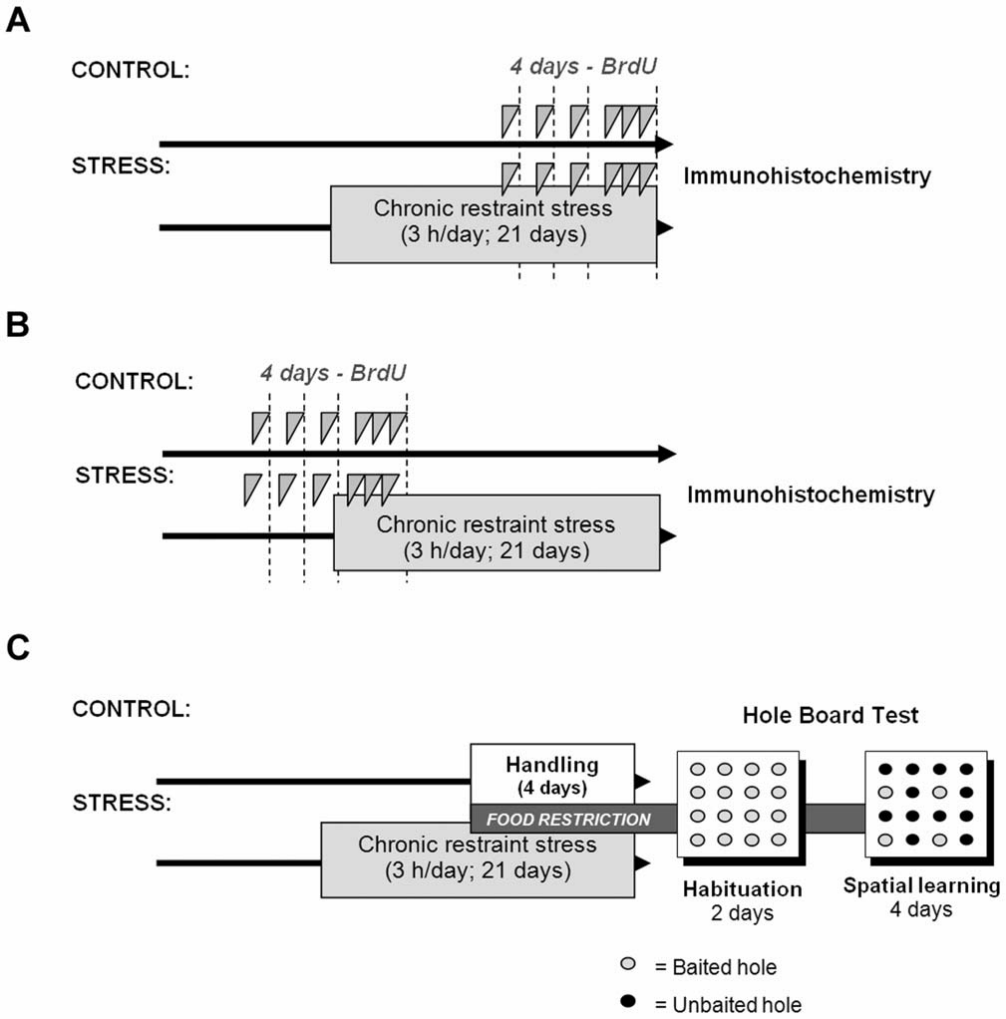
BrdU administration and behavioural analysis protocols. Analyses of new hippocampal cell proliferation (A) survival (B) and behaviour on the hole-board test (C) were carried out in different groups of mice from both genotypes submitted either to standard housing (control) or 21 days of chronic stress. (A) To assess cell proliferation, BrdU injections (75 mg/kg) were given on the final 4 days of stressor exposure in the chronically stressed groups. All animals were sacrificed by perfusion the day following the final BrdU dose. Brain tissue from these animals was also processed for PCNA, DCX and GFAP immunolabeling and apoptosis. (B) For cell survival assessments, BrdU was administered on the 4 days prior to stressor exposure in the chronically stressed groups, and all animals were sacrificed by perfusion 22 days following the final BrdU injection. These animals were also assessed for NeuN staining. (C) Behavioural analyses were conducted using the hole-board test over 2 days of habituation and 4 days of spatial learning. All holes were baited during habituation, but only 4 were baited during the spatial learning phase.

Animals of both genotypes were randomly submitted to either the control or chronic stress treatment. Six to 8 animals for each of the 4 experimental conditions (control-WT, chronic stress-WT, control-NULL, chronic stress-NULL) were used to assess the following properties of the hippocampus: cell proliferation; the number of neural progenitors; the phenotype of new cells; apoptosis; and the population of young neurons (Figure 1A). Another group of 6–8 animals per experimental condition was used to assess the effects of stress and the absence of LPA_1_ on the survival of new cells and hippocampal structure (Figure 1B).Basal corticosterone levels were assessed in another group of 5–7 animals per experimental condition and genotype. Finally, a separate group of WT and NULL mice underwent control or chronic stress treatment (9–12 per experimental condition) and were used for behavioural assessment on the hole-board test (Figure 1C).

### Chronic stress protocol

Mice in the chronically stressed groups were restrained 3 h daily for 21 consecutive days in a modified 50 ml, clear polystyrene conical centrifuge tube with multiple air holes for ventilation. Restraint began at 10:00 a.m. each day. Control mice remained undisturbed in their home cages.

### Histological procedures

#### BrdU administration paradigm

Administration of bromodeoxyuridine (BrdU, Sigma, St. Louis, USA) consisted of a daily intraperitoneal injection (75 mg/kg, dissolved in saline) for 3 days, and 3 additional doses on the fourth day, with an injection every 3 h (Figure 1A,B) [34]. For assessment of cell proliferation, animals were sacrificed by perfusion the day following the final BrdU dose. Injections were given on the final 4 days of stressor exposure in the chronically stressed groups (Figure 1A). For cell survival assessments, mice were sacrificed by perfusion 22 days following the final BrdU injection. In these cases, BrdU administration took place on the 4 days prior to stressor exposure for the chronically stressed groups (Figure 1B). The effects of stress on hippocampal cell proliferation and survival were assessed independently using this protocol.

#### Immunohistochemistry and apoptosis determination

Animals were transcardially perfused with 0.1 M phosphate-buffered saline (PBS) and a periodate-lysine-paraformaldehyde (PLP) solution in PBS [39]. Brains were removed and post-fixed for 48 h in PLP at 4°C. Immunohistochemistry was performed as described in [34] on free-floating vibratome hippocampal coronal sections (50 µm). BrdU immunolabelling required a 15 minutes digestion in a solution of 5 µg/ml proteinase K (Sigma, St. Louis, USA), followed by denaturation of the DNA in 2N HCl for 30 minutes at 37°C and subsequent neutralisation in 0.1 M boric acid (pH 8.5). Sections were incubated overnight in the following antibodies: mouse anti-bromodeoxyuridine (BrdU; 1∶1000; DSHB, University of Iowa, USA), mouse anti-Proliferating Cell Nuclear Antigen (PCNA; 1∶1000; Sigma), goat anti-doublecortin (DCX; 1∶200; Santa Cruz Biotechnology, Santa Cruz, USA), rabbit anti-Glial Fibrillary Acid Protein (GFAP; 1∶1000; Dako, Glostrup, Danmark), or mouse anti-neuron-specific nuclear protein (NeuN; 1∶500; Chemicon, Temecula, USA). Standardised detection was performed using biotin-conjugated rabbit anti-mouse, rabbit anti-goat or swine anti-rabbit (as appropriate) immunoglobulins (Dako), ExtrAvidin®-peroxidase (Sigma) and DAB (Sigma). In animals in which BrdU was injected to mark cell proliferation, sections stained for DCX or GFAP were double-labelled with BrdU using DAB with nickel chloride (Sigma) for intensification. Apoptosis determination was performed with an in situ apoptosis detection kit (NeuroTACS-II, Trevigen, Gaithersburg, USA) following the manufacturer's instructions.

#### Cell quantification

Cell counting was performed with an Olympus BX51 microscope equipped with an Olympus DP70 digital camera at 100× magnification (Olympus, Glostrup, Denmark). Cells were counted in the granular zone and SGZ of the DG. For apoptosis measurement, cells were counted in each layer of CA1 (oriens, pyramidal, radiatum), CA3 (oriens, pyramidal, radiatum) and the DG (molecular, granular, hilus). Layers were determined according to anatomical criteria [40].

Quantification of the number of BrdU+ (injected to examine proliferation or survival), PCNA+, DCX+, or apoptotic cells was carried out using a modified stereological method based on Kempermann et al. [41], [42] and others [43]. Briefly, the total number of positive cells was counted in 1 of every 4 (for BrdU+ cells) or 1 of every 8 (PCNA+, DCX+ and apoptotic cells) equally spaced hippocampal sections. Data were multiplied by 4 or 8 as appropriate to estimate the total number positively stained cells per hippocampus.

To study the phenotype of new cells, the number of proliferating BrdU+ cells that co-labelled with DCX (neuronal phenotype) or GFAP (with stellar morphology, indicating astrocyte phenotype) [3] were counted in 1 of every 8 equally spaced hippocampal sections double-stained for BrdU and DCX or GFAP. The percentage of BrdU-labelled cells positive for the phenotype marker was calculated for each section, and a mean among sections was obtained for each animal. Quantification of the total number of primary neural progenitor cells was also performed using GFAP-stained sections. These cells were located in the SGZ and were defined by GFAP+ staining together with radial morphology [44], .

#### Maturation of DCX+ cells

The maturation of young hippocampal neurons was assessed by two means. First, DCX+ cells in 1 of every 8 hippocampal sections were classified into two populations as previously reported [46], [47]. The less mature population (referred to as A–D cells) corresponded to DCX+ cells with absent or short dendritic processes, or processes parallel to the SGZ. Thus, the more mature population (referred to as E–F cells) consisted of cells with at least one vertical apical dendrite that penetrated the granule cell layer. The percentage of less mature, DCX+ cells (A–D) was calculated for each section, and a mean among sections was obtained for each animal.

To examine the dendritic arborisation of the more mature population of DCX+ cells (E–F) [47], a representative photo (340×340 µm) of the upper blade of the DG from each section was taken at 40× magnification. Photos were superimposed onto a matrix of horizontal parallel lines spaced 40 µm apart. The number of dendrites crossing each line was counted for every DCX+ cell with E–F morphology in the image.

#### Hippocampal volume and neuronal density in the granule cell layer

Hippocampal volume analysis was performed in 1 of every 4 consecutive NeuN-stained hippocampal sections using the CAST-Grid software package (Olympus). The hippocampus was divided into CA1, CA3 and DG regions. These regions were subsequently subdivided into each of their cellular layers according to the criteria of Paxinos and Franklin [40]. These layers were analysed separately. The volume (mm^3^) of each layer was estimated with Cavalieri's principle: *Est*_(Vref)_ = *T* × (a/p) × Σ*P*, where *T* represents the mean distance between the consecutively counted sections, (a/p) refers to the area associated with each point of a grid generated over each tissue section by the CAST-Grid system (12763 µm^2^, corrected for the magnification of the image) and *P* is the number of points counted within each area of the hippocampus [48]. Cavalieri's coefficient of error (*CE*) of the volume of any subregion was calculated as follows: *CE* (Σ*P*) = √[(3*A* + *C* - 4*B*)/12]/Σ*P,* where *A* = Σ*P*i^2^, *B* = Σ*P*i *P*_(i+1)_ and *C* = Σ*P*i *P*_(i+2)_ [48], [49]. To estimate the density of NeuN+ cells in the DG granular layer, a random set of sampling frames (10.82×10.82 µm) was generated for each section using the CAST-Grid software package. In each animal, NeuN+ nuclei were counted in 10 µm (beginning 3 µm below the surface). At least 100 frames were analysed for NeuN in this way (representing 5% of the entire analysed area). Neuronal density (NeuN+/µm^3^) was calculated using the following formula: *N*v = Σ(*Q*−)/Σ(*ha*_fra_), where *Q*− is the total number of NeuN+ nuclei counted in all examined frames, *a*_fra_ is the area of the sampling frames used and *h* is the thickness of the sections from which NeuN+ nuclei were counted [50]. The total number of neurons was calculated for each animal by multiplying the neuronal density (NeuN+/mm^3^) by the volume (mm^3^).

### Corticosterone assay

Mice from both genotypes were rapidly decapitated at 12:00 a.m., and trunk blood was collected in tubes containing EDTA. The tubes were centrifuged, and the supernatant stored at −80°C. Control mice were taken directly from their home cage and sacrificed immediately, whereas chronically stressed mice were sacrificed the day following the completion of the chronic stress treatment. Serum corticosterone concentrations were determined, in duplicate, using a commercially available radioimmunoassay kit for corticosterone analysis (*DPC-Coat-A-Count kit,* Diagnostic Products Corporation, Los Angeles, USA), following the manufacturer's instructions. The intra-assay variability was less than 8%.

### Spatial learning in the hole-board

The effect of chronic stress on spatial memory in WT and maLPA_1_-null mice was examined with the hole-board test. The hole-board test is a widely used spatial memory task that we have shown to be adequate for simultaneously assessing exploration, anxiety-like behaviour and spatial reference and working memory in maLPA_1_-null mice [35].

Chronically stressed groups underwent the test one day following the completion of the stress procedure, whereas mice from the control groups were handled for 1 week prior to the test (5 minutes per day) to allow them to habituate to the experimenter. All groups were food-deprived for 4 days prior to behavioural testing so that their body weights were reduced to 80–85% of their free-feeding weights. Food restriction lasted through the behavioural experiment (Figure 1C). Behavioural testing on the hole board (40×40 cm, containing 16 equidistant holes) was performed between 9:00 a.m. and 3:00 p.m. over 2 days of habituation (1 daily session of 3 minutes) and over 4 days of spatial learning (2 sessions of trials each day with an inter-session interval of 2 h) (Figure 1C; see [35] for a detailed description of the behavioural protocol).

Locomotion (mm travelled per second), thigmotaxis (percentage of time in the periphery, defined as the area within 6.5 cm of the walls) and head dipping (number of hole visits per minute) were registered using a video tracking system (Ethovision XT, Noldus, Wageningen, The Netherlands) and were also recorded by an observer who watched the video. Locomotion and head dipping were expressed as rates per unit of time because they were intended as measures of exploratory activity and not of task accuracy. The reference memory ratio was defined as the number of visits and revisits to the baited holes divided by the total number of hole visits (visits and revisits to the baited and non-baited holes). The working memory ratio was expressed as the number of food-rewarded visits divided by the number of visits and revisits to the baited holes [51]. Importantly, differences in motivation to eat were ruled out because all mice ate normally when food was placed in their home cages.

### Statistical analyses

Data from histological markers and corticosterone measurements were analysed using a two-way ANOVA (‘genotype × stress’) followed by a post hoc Fisher Least Significant Difference test (LSD). Data from the hole-board test (averaged as a single daily score per animal) and from the dendrite length quantification analysis were analysed using a three-way repeated measures ANOVA (‘genotype × stress × day’ or ‘genotype × stress × distance from soma’, respectively) followed by a LSD test. The relationship among the measures assessed for spatial learning (averaged to 1 score per animal) was assessed separately for each group using Pearson correlations. Only probabilities less than or equal to .05 were considered significant.

## Results

### The LPA_1_ receptor modulates the effects of chronic stress on hippocampal cell proliferation, apoptotic cell death and new neurons number and maturation

#### Cell proliferation and neural progenitors

**Figure 2:**
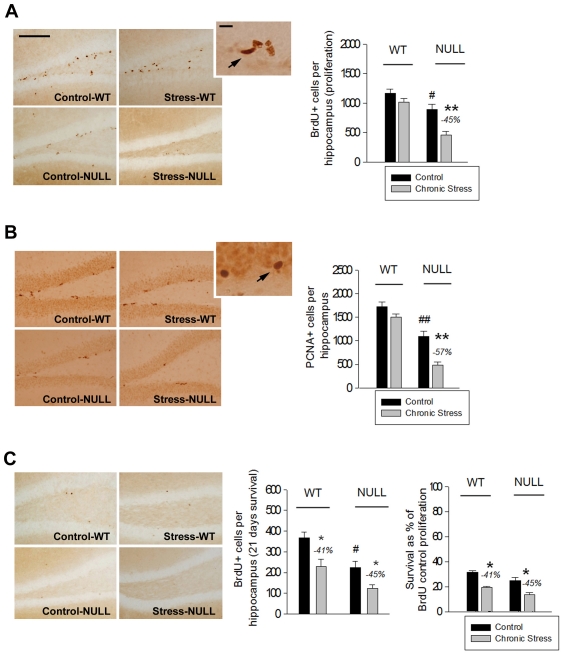
Cell proliferation and survival in the dentate gyrus. Mean values ± SEM. (A, B) MaLPA_1_-null mice showed defective cell proliferation as assessed by BrdU (A) and PCNA (B) immunolabeling. Notably, the number of proliferating cells was reduced by chronic stress only in the mutant genotype. (C) Chronic stress reduced the survival of new cells labelled with BrdU prior to stress treatment, but cell survival was similar between genotypes when data were corrected for their distinct proliferation rates. (- %): reduction from the control group. Scale bar in A (also valid for B and C): 100 µm; 20 µm. Arrows indicate cells positive for BrdU (A) and PCNA (B). Post-hoc LSD tests: (*p<.05; **p<.001): significant difference between the stressed group vs. its control; (#*p*<.05; ##*p*<.001): significant difference between the control NULL vs. the control WT mice.

Hippocampal cell proliferation was examined by means of BrdU and PCNA immunolabeling. Analysis of BrdU-labelled cells using two-way ANOVAs followed by LSD post-hoc analysis showed less hippocampal proliferation in maLPA_1_-null mice. Moreover, chronic stress reduced cell proliferation only in the absence of the LPA_1_ receptor (Figure 2A). The assessment of the endogenous proliferation marker PCNA [52] also indicated defective proliferation in the NULL mice. In addition, PCNA+ cells were dramatically reduced by chronic stress only in the mutant genotype (Figure 2B), confirming results obtained with BrdU. The population of neural progenitor cells was similar in WT and maLPA_1_-null mice and was equally reduced in both genotypes following stress. This finding indicates that damage in this cell pool was unlikely to be responsible for the proliferation differences found between groups.

ANOVA results: BrdU (proliferation): ‘genotype’: *F*(1,20) = 27.060, *p* = .000; ‘stress’: *F*(1,20) = 17,644, *p* = .000; ‘genotype × stress’: *F*(1,20) = 4.830, *p* = .040; LSD test shown in Figure 2A. PCNA: ‘genotype’: *F*(1,20) = 79.186, *p* = .000; ‘stress’: *F*(1,20) = 20.216, *p* = .000; ‘genotype × stress’: *F*(1,20) = 4.745, *p* = .042; LSD test shown in Figure 2B. Neural progenitor cells: ‘stress’: *F*(1,20) = 53.250, *p* = .000; LSD: *p* = .000; number of neural progenitor cells per hippocampus expressed as mean ± SEM: control WT: 3059±144; stressed WT: 2154±96; control NULL: 2908±96; stressed NULL: 1933±168.

#### Cell survival

The effect of stress on a new cell’s long-term survival was assessed 21 days following BrdU administration, in cells labelled prior to the onset of the chronic stress protocol. WT mice had a greater total number of surviving cells (Figure 2C). However, survival was similar in both genotypes when it was calculated as a percentage of the reduction from each genotype’s proliferation baseline (i.e., the mean proliferation of the control WT or NULL groups assessed by BrdU immunolabelling, Figure 2C). Importantly, chronic stress reduced the survival of new cells equally in both groups (Figure 2C).

ANOVA results: ‘genotype’: F(1,23) = 15.603, *p* = .001; ‘stress’: F(1,23) = 14.339, *p* = .001; LSD test shown in Figure 2C.

#### Neuronal and glial phenotypes

**Figure 3:**
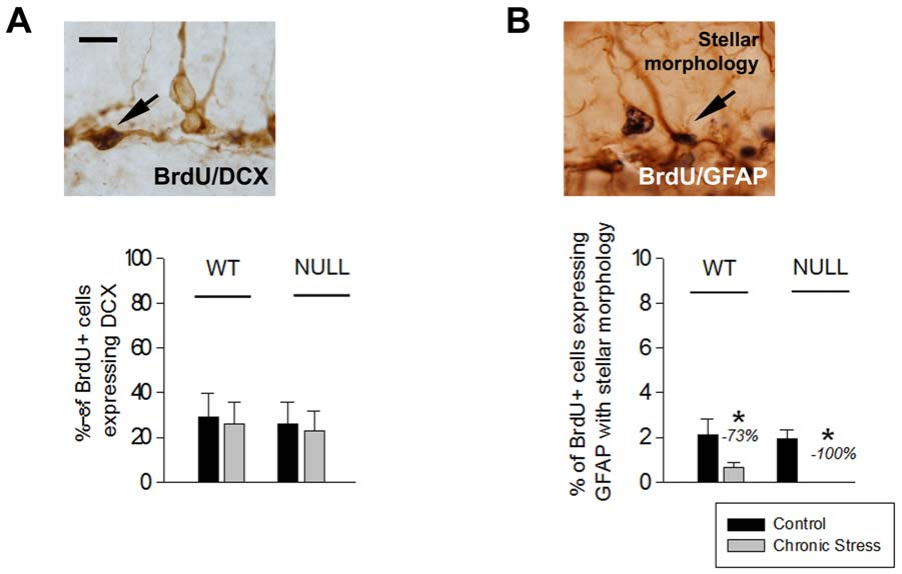
Neuronal and glial phenotype of the newly born hippocampal cells. Mean values ± SEM. The absence of LPA_1_ receptor did not affected the percentage of BrdU labelled cells (aged 1 to 4 days) that had differentiated into immature neurons (A, BrdU co-labelling with DCX) or in astrocytes (B, BrdU co-labelling with a GFAP+ cell with stellar morphology), although chronic stress reduced the glial fate. (- %): reduction from the control group. Scale bar in A (also valid for B): 10 µm. Post-hoc LSD tests: (*p<.05): significant difference between the stressed group vs. its control.

The analysis of differentiation of newly born cells into young neurons (i.e. the percentage of BrdU+ cells aged 1 to 4 days that were DCX+) revealed no effects of stress nor genotype and suggested no alterations in neuronal fate determination (Figure 3A). Chronic stress reduced the percentage of new cells that differentiated into astrocytes (i.e. the percentage of BrdU+ cells aged 1 to 4 days that were GFAP+ with stellar morphology). No difference in this effect, however, was observed between genotypes (Figure 3B).

ANOVA results: Glial phenotype: ‘stress’: *F*(1,20) = 15.061, *p* = .001; LSD test shown in Figure 3B.

#### Apoptosis

**Figure 4:**
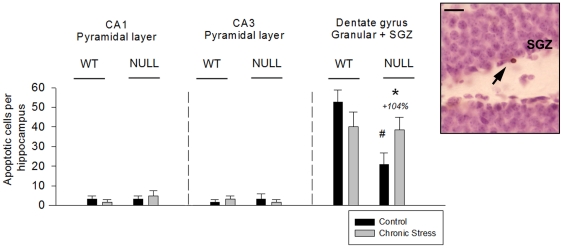
Apoptosis in the cellular layers of the hippocampus. Mean values ± SEM. In the granular and subgranular zones (SGZ) of the dentate gyrus, maLPA_1_-null mice showed reduced apoptosis in the control condition, although apoptosis was increased following chronic stress. Interestingly, apoptotic cells in this layer were frequently located inside or near the SGZ (arrow). In non-proliferative zones of the hippocampus, such as the CA1 and CA3 pyramidal layers, apoptotic nuclei were scarce and not affected by stress or genotype. (+ %): increase from the control group. Scale bar: 20 µm.Post-hoc LSD tests: (*p<.05): significant difference between the stressed group vs. its control; (#*p*<.05): significant difference between the control NULL vs. the control WT mice.

The number of apoptotic cells counted in the hippocampal granular layer, including the SGZ, revealed decreased basal apoptosis in the control NULL mice. However, this was the only genotype in which apoptosis was increased following chronic stress (Figure 4). Interestingly, apoptotic nuclei in all experimental groups were primarly located in the SGZ or in the inner border of the granular layer, suggesting a probable association with new cells [53]. In contrast, hippocampal apoptosis in cellular layers outside the granular/SGZ (CA1 and CA3 pyramidal cell layers, Figure 4) and in non-cellular layers (data not shown) was scarce and did not reveal an effect of genotype, stress, or an interaction between these variables.

ANOVA results: ANOVA results: Granular + SGZ: ‘genotype’: *F*(1,20) = 9.241, *p* = .008; ‘genotype × stress’: *F*(1,15) = 7.765, *p* = .014; LSD test shown in Figure 4.

#### Number and maturation of DCX+ cells

**Figure 5:**
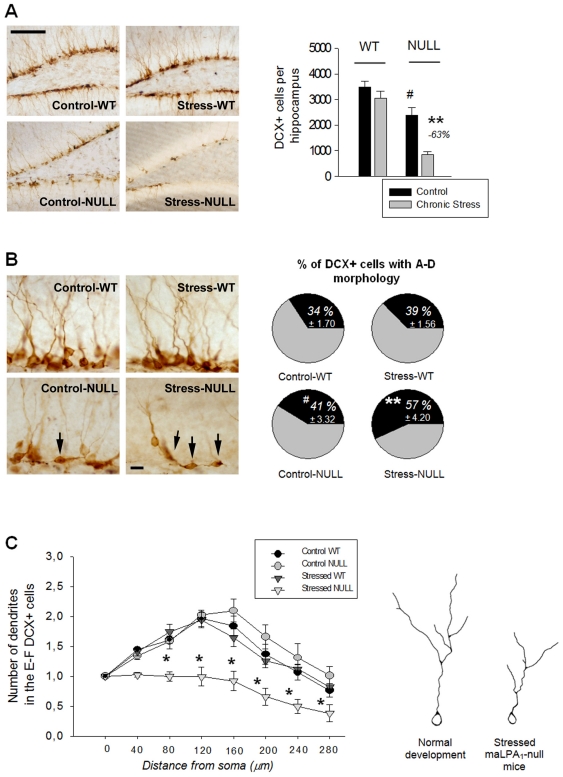
Number and maturation of DCX+ neurons. Mean values ± SEM. (A) The total number of young, DCX+ neurons was reduced in the absence of the LPA_1_ receptor. Chronic stress decreased the DCX+ population only in the mutant genotype. (B) Chronic stress increased the proportion of DCX+ cells that remained in the more immature stages of development (A–D cells, arrows) in mice lacking the LPA_1_ receptor. (C) Moreover, the dendritic arborisation in the more mature DCX+ cells (E–F) was less developed in stressed null mice. A representative drawing is shown next to the graph. (- %): reduction from the control group. Scale bar in A: 100 µm; B: 20 µm. Post-hoc LSD tests: (*p<.05; **p<.001): significant difference between the stressed group vs. its control; (#*p*<.05): significant difference between the control NULL vs. the control WT mice.

The population of young, immature neurons labelled with DCX was decreased in control NULL mice and was further reduced following stress. This population, however, was not impaired in stressed WT mice (Figure 5A). The absence of the LPA_1_ receptor also impaired the maturation of the DCX+ cells. The percentage of less mature DCX+ cells (A–D cells) was greater in the NULL genotype, and this population was increased by stress only in mutants (Figure 5B). Moreover, DCX+ cells that were in a later stage of maturation (E–F cells) demonstrated a less arborised dendritic tree in the stressed NULL genotype (Figure 5C).

ANOVA results: Number of DCX+ cells: ‘genotype’: *F*(1,20) = 46.759, *p* = .000; ‘stress’: *F*(1,20) = 16.307, *p* = .001; ‘genotype × stress’: *F*(1,20) = 5,150, *p* = .034; LSD test shown in Figure 5A. Percent of A–D DCX+ cells: ‘genotype’: *F*(1,20) = 22.767, *p* = .000; ‘stress’: *F*(1,20) = 16.338, *p* = .000; ‘genotype × stress’: *F*(1,20) = 4.431, *p* = .044; LSD test shown in Figure 5B. Dendritic development of E–F DCX+ cells: ‘genotype’: *F*(1,20) = 7.625, *p* = .011; ‘stress’: *F*(1,20) = 20.195, *p* = .000; ‘genotype × stress’: *F*(1,20) = 18.804, *p* = .000; ‘distance from soma’: *F*(7,140) = 56.734, *p* = .000; ‘distance × genotype’: *F*(7,140) = 6.340, *p* = .000; ‘distance × stress’: *F*(7,140) = 2.657, *p* = .0127; ‘genotype × distance × stress’: *F*(7,140) = 3.717, *p* = .001; LSD test shown in Figure 5C.

#### Hippocampal volume and neuronal density in the granular layer

The structure of the hippocampus was analysed using sections stained with the mature neuronal marker NeuN This analysis revealed decreased volumes in the CA1 and CA3 areas in maLPA_1_-null mice (Table 1). Stress equally reduced the volume of the pyramidal layer in both genotypes, but the volume of the granular layer of the DG was only reduced in the stressed NULL genotype (Table 1). The CE ranged from .05 to .10 in all cases (mean: .08). To complement these results, we stereologically estimated the density and total number of NeuN+ cells in the granular cell layer, finding that stressed NULL mice exhibited a lower density and a reduced total number of mature NeuN+ neurons (Table 1).

**Table 1:** Hippocampal structure in control and stressed WT and maLPA_1_-null mice in NeuN-stained sections.

Volume of hippocampal layers (mm^3^)				
	Control WT	Stressed WT	Control NULL	Stressed NULL
***CA1***				
*Oriens:*	.138±5.046e-3	.132±4.161e-3	.116±5.091e-3 #	.115±5.251e-3
*Piramidal:*	.065±4.560e-3	.057±4.010e-3 *	.056±4.847e-3 #	.037±2.310e-3 *
*Radiatum:*	.293±0.011	.279±0.010	.245±0.011 #	.230±7.553e-3
***CA3***				
*Oriens:*	.098±6.631e-3	.099±4.228e-3	.084±2.926e-3 #	.077±4.083e-3
*Piramidal:*	.068±5.654e-3	.057±3.516e-3 *	.058±3.484e-3 #	.046±3.585e-3 *
*Radiatum:*	.097±4.033e-3	.096±2.715e-3	.079±1.965e-3 #	.073±3.066e-3
***DG***				
*Molecular:*	.172±9.028e-3	.167±7.372e-3	.162±5.858e-3	.160±7.168e-3
*Granular:*	.063±3.614e-3	.057±2.204e-3	.064±2.815e-3	.046±1.538e-3 *
*Hilus:*	.032±1.308e-3	.031±1.537e-3	.032±2.815e-3	.031±1.626e-3
***Total***	1.026±0,815	.975±0,030	.896±0,028 #	.815±0,031

Cell density (NeuN+/µm^3^) and total number of mature neurons NeuN+ in the dentate gyrus’ granular cell layer				
	Control WT	Stressed WT	Control NULL	Stressed NULL
***Density***	.370e-2±217e-6	.386e-2±258e-6	.377e-2±221e-6	.309e-2±115e-6 *
***Total number***	241e3±290e2	205e3±186e2	234e3±223e2	143e3±581e2 *

Mean values ± SEM. MaLPA_1_-null mice showed reduced hippocampal volume in the oriens, pyramidal and radiatum layers in the CA1 and CA3 areas; no structural alterations were observed in the DG. Chronic stress reduced the volume of the pyramidal layer in both genotypes; however, reduced DG granular layer volume was observed only in the stressed NULL genotype, implying a reduction in the density and total number of NeuN+ neurons. Scale bars: 100 µm, 5 µm. Post-hoc LSD tests:

(*p<.05): significant difference between the stressed group vs. its control;

(#*p*<.05): significant difference between the control NULL vs. the control WT mice.

ANOVA results: Volume: Total: ‘genotype’: *F*(1,27) = 19.690, *p* = .000; ‘stress’: *F*(1,27) = 4.027, *p* = .050; CA1 oriens: ‘genotype’: *F*(1,27) = 15.223, *p* = .000; CA1 pyramidal: ‘genotype’: *F*(1,27) = 12.186, *p* = .002; ‘stress’: *F*(1,27) = 10.145, *p* = .004; CA1 radiatum: ‘genotype’: *F*(1,27) = 21.853, *p* = .000; CA3 pyramidal: ‘genotype’: *F*(1,27) = 5.723, *p* = .024; ‘stress’: *F*(1,27) = 6.617, *p* = .016; CA3 radiatum: ‘genotype’: *F*(1,27) = 43.908, *p* = .000; DG granular: ‘stress’: *F*(1,27) = 20.455, *p* = .000; ‘genotype × stress’: *F*(1,27) = 3.471, *p* = .049. Neuronal density of the granular cell layer: ‘genotype’: *F*(1,27) = 4.495, *p* = .048; ‘genotype × stress’: *F*(1,27) = 4.129, *p* = .046. Total number of neurons in the granule cell layer: ‘stress’: *F*(1,27) = 10.613, *p* = .004; ‘genotype × stress’: *F*(1,27) = 4.615, *p* = .046. LSD tests listed in Table 1.

### Chronic stress induced hypocortisolemia in LPA_1_ null mice

**Figure 6:**
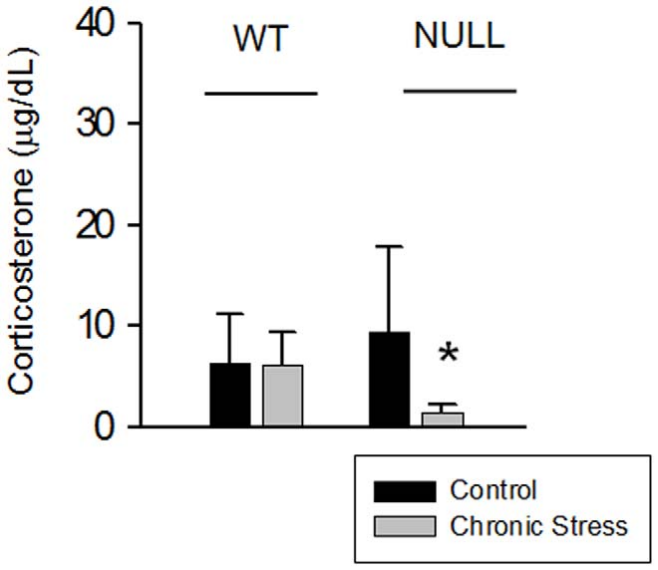
Basal corticosterone levels in control and chronic stress conditions. Mean values ± SEM. Basal serum corticosterone levels were not altered in normal mice following chronic restraint stress, but chronically stressed null mice exhibited hypocortisolemia. Post-hoc LSD tests: (*p<.05): significant difference between the stressed group vs. its control.

Analysis of basal serum corticosterone levels did not show any significant difference between the genotypes in control conditions (Figure 6). However, reduced corticosterone levels were observed in NULL mice after chronic stress. Corticosterone levels in chronically stressed WT mice were no different from those of mice in the control condition (Figure 6).

ANOVA results: ‘stress’: *F*(1,19) = 4.900, *p* = .039; ‘genotype × stress’: *F*(1,19) = 4.700, *p* = .043. LSD test shown in Figure 6.

### Spatial reference memory impairment induced by chronic stress is aggravated by the absence of the LPA_1_ receptor

#### Spatial reference and working memory performance

**Figure 7:**
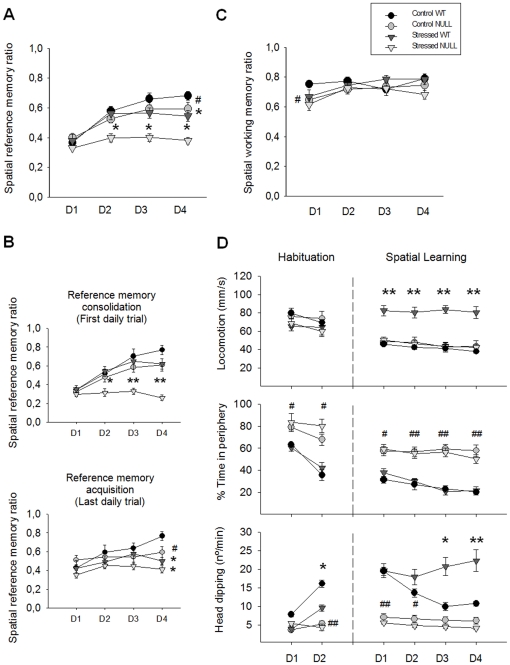
Spatial memory and exploratory and anxiety-like behaviour on the hole-board test. Mean values ± SEM. (A) Chronic stress impaired reference memory in both genotypes, but the deficit was more dramatic in maLPA_1_-null mice. (B) This differential impairment was related to memory consolidation rather than acquisition, although acquisition was also affected by stress. (C) Chronic stress did not impair working memory, but null groups performed worse on the first training day. (D) MaLPA_1_-null mice spent more time in the periphery (thigmotaxis) throughout the testing procedure. Only WT mice showed hyperactivity (locomotion, head dipping) in response to stress. D: Training day. Post-hoc LSD tests: (*p<.05; **p<.001): significant difference between the stressed group vs. its control; (#*p*<.05; ##*p*<.001): significant difference between the control NULL vs. the control WT mice.

Three-way repeated-measure ANOVAs performed for the 4 days of spatial learning confirmed the role of the LPA_1_ receptor in spatial reference memory (Figure 7A). Importantly, chronic stress treatment differentially altered reference memory in both genotypes, with a more dramatic impairment observed in the stressed nulls (Figure 7A). To further investigate reference memory, the first and final daily trials were analysed separately, as these performances represent long-term memory consolidation and short-term acquisition, respectively. In both cases, chronic stress impaired reference memory; interestingly, however, the greater effect of stress on the NULL genotype (‘genotype × stress’ interaction) was only significant when considering the first trials of the day (Figure 7B). Analyses revealed differences in working memory between genotypes, but chronic stress treatment had no effect on this function (Figure 7C). Both control and stressed NULL mice had poorer working memory than control WT mice on the first training day, although both groups were able to improve in training (Figure 7C).

ANOVA results: Spatial reference memory: ‘genotype’: *F*(1,39) = 20.740, *p* = .000; ‘stress’: F(1,39) = 27.639, *p* = .000; ‘day’: *F*(3,117) = 42.031, *p* = .000; ‘genotype × stress’: *F*(1,39) = 5.145, *p* = .029; ‘genotype × day’: *F*(3,117) = 5.568, *p* = .009; ‘stress × day’: *F*(3,117) = 8.331, *p* = .000; LSD test shown in Figure 7A. Spatial reference memory on first daily trials: ‘genotype’: *F*(1,39) = 32.703, *p* = .000; ‘stress’: *F(*1,39) = 13.926, *p* = .000; ‘day’: *F*(3,117) = 22.425, *p* = .000; ‘genotype × stress’: *F*(1,39) = 10.441, *p* = .003; ‘genotype × day’: *F*(3,117) = 3.428, *p* = .025; ‘stress × day’: *F*(3,117) = 4.728, *p* = .004; LSD test shown in Figure 7B. Spatial reference memory on last daily trials: ‘genotype’: *F*(1,39) =  7.043, *p* = .012; ‘stress’: *F*(1,39) =  13.284, *p* = .003; ‘day’: *F*(3,117) = 6.242, *p* = .001; LSD in Figure 7B. Spatial working memory: ‘genotype’: *F*(1,39) = 9.493, *p* = .040; ‘day’: *F*(3,117) = 5.083, *p* = .002; LSD test shown in Figure 7C.

#### Exploratory and anxiety-like behaviour

Locomotion rate, thigmotaxis, and head dipping were assessed through spatial learning and the previous habituation phase. Chronic stress differentially affected both genotypes with respect to locomotion and head dipping, but only WT mice demonstrated increases in these behaviours following stress [54], [55] (Figure 7D). Therefore, NULL mice were unable to increase their activity even following chronic stress treatment. NULL mice spent more time in the periphery than WT mice but no effect of stress on thigmotaxis was found (Figure 7D).

ANOVA results: Locomotion: ‘genotype’: *F*(1,39) = 11.792, *p* = .001; ‘stress’: *F*(1,39) = 9.694, *p* = .003; ‘day’: *F*(3,117) = 15.592, *p* = .000; ‘genotype × stress’: *F*(1,39) = 22.591, *p* = .000; ‘stress × day’: *F*(3,117) = 13.217, *p* = .000; ‘genotype’ × day’: *F*(3,117) = 4.366, *p* = .000; ‘genotype’ × stress × day’: *F*(3,117) = 4.768, *p* = .000. Percent of time in periphery: ‘genotype’: *F*(1,39) = 148.460, *p* = .000; ‘day’: *F*(3,117) = 47.776, *p* = .000; ‘genotype’ × day’: *F*(3,117) = 3.464, *p* = .005. Head dipping: ‘genotype’: *F*(1,39) = 104.26, *p* = .000; ‘day’: *F*(3,117) = 15.747, *p* = .000; ‘genotype’ × day’: *F*(3,117) = 10.136, *p* = .000; ‘stress × day’: *F*(3,117) = 4.917, *p* = .000; ‘genotype’ × stress × day’: *F*(3,117) = 8.2037, *p* = .000. LSD tests shown in Figure 7D.

#### Relationship among the assessed variables

Direct Pearson’s correlations were found between locomotion and head dipping measures (control WT: .73; stressed WT: .85; control NULL: .86; stressed NULL: .80; *p*<.05), suggesting a common dimension of activity and exploration. Importantly, thigmotaxis never correlated with any other variable (*p*>.05). A number of negative correlations (*p*<.05) were identified between memory and activity measures (control WT: reference memory-head dipping (-.67), working memory-locomotion (-.59), working memory-head dipping (-.70); stressed WT: reference memory-head dipping (-.70), reference memory-locomotion (-.90); stressed NULL: reference memory-locomotion (-.66)), indicating more rapid maze exploration in the mice that least effectively learned the reward’s location.

## Discussion

New cells are constantly proliferating in the SGZ of the DG in the adult hippocampus, primarily giving rise to new hippocampal neurons [1], [3]. In accordance with our previous observations [34], in this study, we show that deletion of the LPA_1_ receptor leads to a significant reduction in adult hippocampal cell proliferation (BrdU+ and PCNA+ cells). Interestingly, no structural abnormalities (volume, NeuN+ cell density, pool of precursor cells) or vascular alterations [34] were observed in the DG of maLPA_1_-null mice. Thus, their proliferative deficit may be linked to the absence of the LPA_1_ receptor in adulthood and not to defective neurodevelopment. In contrast, chronic stress is an external factor that alters the neurogenic niche and constitutes a potent modulator of adult neurogenesis [10]. Nevertheless, the specific consequences of chronic stress on proliferation in the SGZ appear to strongly depend on the characteristics of the stress protocol employed, such as its intensity, duration, controllability, and predictability [16], [56]–[58] (reviewed in [10], [13]). The results from our chronic stress treatment agree with recent research in which survival, but not proliferation, was significantly affected by chronic restraint in normal mice [13]. The preservation of the proliferative capacity of the DG may reflect a progressive adaptation to a predictable chronic stressor [59], [60]. However, we show that chronic stress critically exacerbates the previous proliferation deficit in maLPA_1_-null mice, demonstrating that a lack of LPA_1_ signalling induces vulnerability to chronic stress, precipitating hippocampal pathology.

As with cell proliferation, apoptosis is a critical factor in regulating adult neurogenesis, because chronic stress promotes the death of hippocampal neural progenitor cells and young and old hippocampal neurons [10], [16], [61]. However, many of the affected cells (e.g., cells marked with BrdU for the assessment of cell survival) may die long before apoptosis detection after 21 days of stress treatment [16], [41], [62]. This possibility may explain the observation that stressed wild-type animals did not increase their apoptotic response in despite of cell loss. In contrast, apoptosis was increased in stressed null mice, but only in the granular and subgranular zones, in which the other experimental groups showed a degree of apoptosis that seemed in accordance with the observed proliferation rate. In this regard, the correlation of apoptosis with cell proliferation in the granular/SGZ is frequently reported [43], [63], [64], to the extent that apoptosis is normally reduced when hippocampal cell proliferation decreases following chronic stress [16]. In this way, the altered pattern of increased apoptosis together with the reduced proliferation observed in stressed nulls, likely involves the widespread death of newer neurons and may have functional consequences for hippocampal-dependent cognition [64]. These mechanisms are in complete agreement with the role of LPA_1_ signalling in the developing nervous system, where receptor loss increases apoptosis, and gain-of-function prevents it [65]–[67]. The role of LPA_1_ in reducing apoptosis [30], [67] could be further required in altered physiological situations such as in chronic stress or serum withdrawal [66].

Altered cell proliferation and apoptosis must not be considered mutually exclusive given that both can contribute to reduced neurogenesis. Impairment in the population of adult-born neurons in maLPA_1_-null mice was confirmed by the decreased number of DCX+ cells, that was drastically reduced following stress. DCX, a reliable marker of adult hippocampal neurogenesis [68], is expressed in early postmitotic neuronal precursors (cells that maintain their mitotic capacity) and continues to be expressed in developing young immature neurons. This expression peaks at 1 week of age and decreases until neurons mature at 3–4 weeks [45], [69]. In this regard, it is also important to note that the absence of the LPA_1_ receptor not only aggravated stress-induced depletion of DCX+ cells but also disrupted their differentiation and dendritic development, likely damaging their functionality [70]. Related to this result, the decreased neuronal density (NeuN+ neurons) and DG volume of stressed null mice may also reflect a reduced number of new neurons that become fully mature and incorporated into the granular zone [69]. However, this hypothesis was not directly assessed in this study; neuronal density may also be affected by the death of older granule neurons, whereas many other factors, such as putative glial changes or a shift in fluid balance, may result in shrinking of the hippocampus [71].

Regardless, recent studies indicate that immature neurons possess unique electrophysiological and plastic properties [8], [72] that are relevant to hippocampal function [4], [64], [73]–[75]. Therefore, their correct connectivity with previously formed hippocampal circuits is crucial for spatial memory [70]. Supporting the idea that impairment in adult-born neurons has functional consequences for hippocampal-dependent memory, spatial reference memory was defective in maLPA_1_-null mice but was critically damaged in stressed nulls. Although spatial reference memory has been closely linked to both mature and immature new hippocampal neurons [4], [73], [74], neurogenesis may be more involved in tasks that require greater hippocampal participation [4], [76]. This involvement occurs when memory must be stored for long periods of time [4], [73], [77], [78], explaining the greater damage to memory consolidation compared to acquisition in stressed null mice. Deficits were specific to reference memory because chronic stress did not differ from previous spatial working memory performance. It is interesting to note that, although dependent on hippocampal function, neither spatial working memory nor the learning of related procedural rules seem to require hippocampal neurogenesis [78], [79]. Moreover, it should be noted that spatial working memory also relies on the medial prefrontal cortex, which may support this cognitive function when the hippocampus is damaged [80]. The reported effects of stress on the medial prefrontal cortex have been contradictory [9], as these consequences are complex and circuit-specific [81]. Finally, results from the hole-board test demonstrated increased thigmotaxis with reduced exploration in the NULL genotype. Although these behavioural alterations may reflect altered anxiety responses [35], [36] and motor deficits [30], [35], [36] in the absence of LPA_1_, they were independent of (and thus unlikely affected by) cognitive performance [35].

Future research will establish the mechanisms by which the LPA_1_ receptor mediates hippocampal neurogenesis and the stress response. Importantly, the possibilities discussed below are not mutually exclusive, as different aspects of neurogenesis may be regulated by independent mechanisms [47], [62]. The LPA_1_ receptor activates several intracellular signalling pathways mediated by Rho, phospholipase C (PLC), Ras and phosphatidylinositol 3-kinase (PI3K) proteins [82], [83] (reviewed in [23]). These pathways modulate the role of the LPA_1_ in numerous cellular responses, such as cell proliferation, differentiation and survival/apoptosis [23], [24], [30], [34], [67], [82], [83]. Interestingly, Rho and PLC signalling provide a potential molecular mechanism for the crosstalk between the LPA_1_ receptor and chronic stress, as these intracellular pathways are involved in the neuronal responses to stress and glucocorticoids [84], [85] and have been linked to stress-induced behavioural alterations [86], [87]. Considering that the LPA_1_ receptor is expressed in immortalised hippocampal progenitor cells and in hippocampal neurons in vivo [31]–[33], LPA_1_ signalling may be directly activated in these cells under chronic stress conditions. Nevertheless, LPA_1_ activation in glial cells may well influence neurons, for instance by triggering neuronal growth factor release [88]. Alternatively, reduced corticosterone concentrations in null mice following chronic stress, in contrast to normal mice, indicate an impaired adaptation of the hypothalamic-pituitary-adrenal (HPA) axis in the absence of the LPA_1_ receptor. Hypocortisolemia is usually attributed to an initial hyper-reactivity of the HPA axis that, when repeatedly activated, may result in an enhanced axis inhibition or in a blunted response of the adrenocorticotropin hormone to corticotrophin releasing factor stimulation [89], [90]. Therefore, our data suggest that the hormonal response to stressors is altered in LPA_1_ receptor null mice, also taking into account that their reduced hippocampal volume and neurogenesis may affect the HPA axis regulation [91]–[93]. However, this hypothesis has not been directly assessed in this study. In any case, glucocorticoids are likely to contribute to the consequences of chronic stress described here, given the fact that they are potent inhibitors of adult hippocampal neurogenesis [94]–[97]. Among other functions, glucocorticoids increase extracellular glutamate levels [98] and crosstalk with glutamatergic receptors [99], actions that have many implications for dendritic restructuring and cell death [96], [97], [100]. Interestingly, an abnormal glutamatergic transmission has been described in the hippocampus of LPA_1_ null mice [101], [102] and may, thus, influence their vulnerability to the action of glucocorticoids on the adult born hippocampal neurons. Lastly, although our results suggest that the involvement of LPA_1_ in the effects of chronic stress is closely related to hippocampal neurogenesis, it should be noted that the exacerbation of other stress-induced hippocampal alterations in the absence of LPA_1_ cannot be ruled out.

Repeated stress is frequent in our society, and its pathological consequences on the hippocampus are extensive and cause emotional and cognitive alterations by impairing adult hippocampal neurogenesis [10], [20], [97], [103], [104]. Defective neurogenesis is hypothesised to represent a core feature in psychiatric disorders, such as major depression and schizophrenia, and may explain the contribution of stress to the development of these pathologies [18], [20], [104]. Here, we provide the first evidence that the LPA_1_ receptor pathway is an important modulator of the effects of chronic stress on adult hippocampal neurogenesis and spatial memory, in addition to its previously reported role under basal conditions [34]–[36]. Therefore, manipulation of the LPA_1_ pathway may have therapeutic potential for the prevention or treatment of hippocampal-dependent cognitive and emotional symptoms via modulation of neurogenesis in stressful conditions, a hypothesis that must be tested in future studies.
