# Correction: Aggravation of Chronic Stress Effects on Hippocampal Neurogenesis and Spatial Memory in LPA_1_ Receptor Knockout Mice

**DOI:** 10.1371/annotation/c105dab8-59b8-467a-9223-5bd92936e49a

**Published:** 2011-11-29

**Authors:** Estela Castilla-Ortega, Carolina Hoyo-Becerra, Carmen Pedraza, Jerold Chun, Fernando Rodríguez De Fonseca, Guillermo Estivill-Torrús, Luis J. Santín

The complete funding information is: "This work was supported by grants from the Spanish Ministry of Education and Science (MEC SEJ2007-61187, MICINN PSI2010-16160 to LJS), Programme for Stabilization of Researchers and Research Technician and Intensification of Research Activity in the National Health System (I3SNS Programme to GET), the Human Frontier Science Programme (to JC, FRDF), Fund for Health Research, Carlos III Health Institute (FIS 02/1643, FIS PI07/0629, FIS PI10/02514 to GET), Red de Trastornos Adictivos RTA (RD06/001 to FRDF), Andalusian Ministry of Health and of Innovation, Science and Enterprise (CTS065 to GET; CTS433 to FRDF) and the US National Institutes of Health MH051699 and MH01723 (to JC). The author E. Castilla-Ortega has a FPU Grant from the Spanish Ministry of Education (AP-2006-02582). The funders had no role in the study design, data collection and analysis, decision to publish, or preparation of the manuscript." 

